# Co-Expression Networks Reveal Potential Regulatory Roles of miRNAs in Fatty Acid Composition of Nelore Cattle

**DOI:** 10.3389/fgene.2019.00651

**Published:** 2019-07-11

**Authors:** Priscila S.N. de Oliveira, Luiz L. Coutinho, Aline S.M. Cesar, Wellison J. da Silva Diniz, Marcela M. de Souza, Bruno G. Andrade, James E. Koltes, Gerson B. Mourão, Adhemar Zerlotini, James M. Reecy, Luciana C.A. Regitano

**Affiliations:** ^1^Embrapa Pecuária Sudeste, Empresa Brasileira de Pesquisa Agropecuária, São Carlos, Brazil; ^2^Department of Animal Science, University of São Paulo, Piracicaba, Brazil; ^3^Department of Agroindustry, Food and Nutrition, University of São Paulo, Piracicaba, Brazil; ^4^Department of Genetics and Evolution, Federal University of São Carlos, São Carlos, Brazil; ^5^Department of Animal Science, Iowa State University, Ames, IA, United States; ^6^Embrapa Informática Agropecuária, Campinas, Brazil

**Keywords:** *Bos indicus*, conjugated linoleic acid, integrative genomics, mRNA, miRNA, oleic acid

## Abstract

Fatty acid (FA) content affects the sensorial and nutritional value of meat and plays a significant role in biological processes such as adipogenesis and immune response. It is well known that, in beef, the main FAs associated with these biological processes are oleic acid (C18:1 cis9, OA) and conjugated linoleic acid (CLA-c9t11), which may have beneficial effects on metabolic diseases such as type 2 diabetes and obesity. Here, we performed differential expression and co-expression analyses, weighted gene co-expression network analysis (WGCNA) and partial correlation with information theory (PCIT), to uncover the complex interactions between miRNAs and mRNAs expressed in skeletal muscle associated with FA content. miRNA and mRNA expression data were obtained from skeletal muscle of Nelore cattle that had extreme genomic breeding values for OA and CLA. Insulin and MAPK signaling pathways were identified by WGCNA as central pathways associated with both of these fatty acids. Co-expression network analysis identified bta-miR-33a/b, bta-miR-100, bta-miR-204, bta-miR-365-5p, bta-miR-660, bta-miR-411a, bta-miR-136, bta-miR-30-5p, bta-miR-146b, bta-let-7a-5p, bta-let-7f, bta-let-7, bta-miR 339, bta-miR-10b, bta-miR 486, and the genes *ACTA1* and *ALDOA* as potential regulators of fatty acid synthesis. This study provides evidence and insights into the molecular mechanisms and potential target genes involved in fatty acid content differences in Nelore beef cattle, revealing new candidate pathways of phenotype modulation that could positively benefit beef production and human consumption.

## Introduction

Fatty acid (FA) content is an important trait that can influence the sensorial and nutritional value of beef and plays a significant role in molecular and physiological processes. Despite that the consumption of beef fatty acids is being associated with metabolic diseases such as type 2 diabetes and obesity effects, such as altered blood lipid and lipoprotein content (Wood et al., 2008), beef has beneficial effects on human health due to its high nutritional value, being also an important source of oleic acid (OA) (Laaksonen et al., 2005a). Likewise, conjugated linoleic acids (CLAs) could have a range of nutritional beneﬁts in the diet (Valsta et al., 2005).

Fatty acid biosynthesis biological processes are complex and dependent on several regulatory mechanisms, such as post-transcriptional regulation of gene expression (Nakamura and Nara, 2003). In this sense, miRNAs have been shown to block the translation of target mRNAs and thereby post-transcriptionally regulate adipogenesis and several other biological processes involved in fatty acid metabolism in bovine (Guo et al., 2017).

Transcriptomic studies have shown that the expression of many miRNAs is species specific and tissue specific, indicating that miRNAs may have potential roles in organ and tissue development, metabolism, immune response (Lawless et al., 2014), milk production traits, and fertility (Fatima and Morris, 2013). In beef cattle, differences in the expression pattern of miRNAs have been identified in animals with different amounts of subcutaneous fat, which could indicate a potential regulatory role of these molecules in the development of adipose tissue (Jin et al., 2010) and fat metabolism (Romao et al., 2014). These studies have identified numerous miRNAs expressed in cattle, but the miRNA regulatory mechanisms that underlie these phenotypes are unclear.

A recent integrative analysis of miRNA–mRNA co-expression in this Nelore population revealed several genes and miRNAs as candidate regulators of intramuscular fat deposition. Glucose metabolism and inflammation processes were the main pathways found to influence intramuscular fat deposition in Nelore beef cattle (Oliveira et al., 2018). Furthermore, previous RNAseq studies including this population identified differences in the skeletal muscle transcriptome profile associated with extreme values of fatty acid content. Oleic acid and CLA-c9t11 content had significant effects on the expression level of genes related to oxidative phosphorylation, cell growth, survival, and migration (Cesar et al., 2016). However, there are no studies about miRNA–mRNA co-expression related to fatty acid composition in bovines.

The integration of previous transcriptomic studies (Cesar et al., 2016) with miRNA expression data information may help provide a better understanding of the molecular mechanisms involved in the variation of FA content and deposition. Therefore, the goal of this study was to perform an integrative miRNA and mRNA expression analysis in skeletal muscle of beef cattle to unravel novel regulatory networks and signaling pathways involved in fatty acid biosynthesis and composition.

## Material and Methods

### Ethics Statement

Experimental procedures were carried out in accordance with the relevant guidelines provided by the Institutional Animal Care and Use Committee Guidelines of the Embrapa Pecuária Sudeste—Protocol CEUA 01/2013. The Ethical Committee of the Embrapa Pecuária Sudeste (São Carlos, São Paulo, Brazil) approved all experimental protocols (approval code CEUA 01/2013) prior to the conduction of the study.

### Phenotypic Data

Description of phenotypic data and genomic heritability for oleic acid (OA (C18:1 cis9) and conjugated linoleic acid (CLA-c9t11) content from Nelore steers were previously reported (Cesar et al., 2014), with genomic heritability mean values of 0.16 ± 0.11 and 0.04 ± 0.09, respectively.

Animals used in the RNAseq and miRNAseq analyses were selected for extreme values of OA and CLA based on the rank of their genomic estimated breeding values (GEBVs) in a larger population of 386 Nellore steers. The GEBVs were calculated by GenSel software using the Bayes B approach (Cesar et al., 2014). A total of 30 animals with extreme GEBVs for OA and CLA content were selected separately: the top 13 high (H-OA) and 15 low (L-OA) animals for OA content, and the top 15 high (H-CLA) and 15 low (L-CLA) animals for CLA content. The Nelore steers used in this study are exactly the same group of animals used in Cesar et al. (2016).

### mRNA Expression Data

The processing and analysis of mRNA expression data from skeletal muscle from the same population of animals used in this study were previously described in Cesar et al. (2016), with the sample accession of mRNA expression data of PRJEB13188. In total, 16,710 genes were identified as expressed in skeletal muscle for OA, and 16,530 genes were expressed in skeletal muscle for CLA. These genes were used for co-expression analysis.

### miRNA Expression Data

The processing and analysis of miRNA expression data from skeletal muscle and miRNA target predictions used in this study followed the same procedures previously described in De Oliveira et al. (2018). Therefore, we will give a brief description of the analyses carried out.

In brief, sequencing of miRNA cDNA libraries was conducted on a MiSeq (Illumina, San Diego, CA) with MiSeq Reagent Kit 50 cycles in the Laboratory Multiuser ESALQ in Piracicaba/SP/Brazil, according to the protocol described by Illumina. The FastQC tools (http://www.bioinformatics.babraham.ac.uk/projects/fastqc) and FASTX (http://hannonlab.cshl.edu/fastx-toolkit) were used to check the quality of reads, and reads were subjected to alignment to bovine genome reference UMD version 3.1 (Ensembl 84: Mar 2016) through the software miRDeep2 version 2.0.0.7 (Friedländer et al., 2008). The reads were then mapped to regions of the genome using the Bowtie tool (Langmead et al., 2009), built into miRDeep2 software.

### Differentially Expressed miRNAs

Differentially expressed (DE) miRNAs were identified for OA and CLA content phenotypes from a total of 30 small RNA libraries derived from skeletal muscle (*N* = 13 H-OA, *N* = 15 L-OA and *N* = 15 H-CLA, *N* = 15 L-CLA) using DESeq2 software (Love et al., 2014). The Benjamini–Hochberg method (Benjamini and Hochberg, 1995) was used to control for the rate of false positive (FDR; 10%) due to the number of genes and miRNAs tested. We set an FDR threshold of 0.1 (i.e., 10% of false positives are expected) to correct for false positives, avoiding to lose information, as these are exploratory analyses that should indicate biological responses to be in the future verified.

#### miRNA Target Predictions and Functional Enrichment Analysis

The target genes of DE miRNAs from skeletal muscle were predicted with TargetScan (Agarwal et al., 2015) and miRanda (Betel et al., 2010) software. In order to predict the potential regulatory target transcripts, the target genes were filtered by skeletal muscle (Cesar et al., 2016) mRNA expression data previously analyzed on the same set of samples. Functional enrichment analysis of target genes was performed by WebGestalt (Wang et al., 2017) using *Bos taurus* and the overrepresentation enrichment analysis (ORA) as organism and method of interest.

### miRNA and mRNA Co-Expression Approaches: Weighted Gene Co-Expression Network Analysis (WGCNA) and Partial Correlation With Information Theory (PCIT)

#### WGCNA

Co-expression networks were constructed by WGCNA (Langfelder and Horvath, 2008) v1.36 package in RStudio environment using miRNA (*N* = 13 H-OA, *N* = 15 L-OA and *N* = 15 H-CLA, *N* = 15 L-CLA) and mRNA (*N* = 13 H-OA, *N* = 15 L-OA and *N* = 15 H-CLA, *N* = 15 L-CLA) skeletal muscle expression data.

miRNA and mRNA networks were constructed separately for high (H) and low (L) fatty acid groups. miRNA network construction and module detection used the step-by-step signed network construction with a soft threshold of β = 6 (*R* ^2^ > 0.90) and a minimum module size of 5. The same approach was adopted for mRNA signed network construction with a soft threshold β = 6 (*R* ^2^ > 0.91) and a minimum module size of 30. Five was chosen as the minimum module size for the miRNAs due to the smaller size of the miRNA transcriptome relative to the mRNA transcriptome (Langfelder and Horvath, 2008; Betel et al., 2010). The topological overlap distance calculated from the adjacency matrix is then clustered with the average linkage hierarchical clustering. The default minimum cluster merge height of 0.25 was retained.

An integrative analysis was performed, in which miRNA module eigengenes (MEs) and mRNA MEs for high and low fatty acid groups were correlated with one another by calculating the Pearson correlations. miRNA and mRNA modules with a negative correlation and a *p*-value < 0.10 were selected for functional enrichment analysis. miRNA modules that were significantly correlated were then further explored to identify hub miRNAs. Hub miRNAs were selected based on the top five greatest module membership (MM) values.

In order to better understand the biological signiﬁcance of the modules identified, the functional enrichment analysis of genes and miRNA target genes were performed by WebGestalt (Wang et al., 2017) web tool. The functional enrichment analysis used the list of target genes from hub miRNAs selected from miRNA modules that were negatively correlated with mRNA modules. Co-expression networks among hub miRNAs and the GO terms of the target genes were constructed in Cytoscape v.3.3.0 (Cline et al., 2007).

#### PCIT: Differential Hubbing (DH), Regulatory Impact Factor (RIF), and Phenotypic Impact Factor (PIF) Metrics

The gene list used for PCIT analyses included all miRNAs and mRNAs detected in our study, but only those with a direct and partial correlation greater than 0.90 were used for the differential hubbing (DH) analyses. The DH was computed by the difference of significant connections of mRNA and miRNA between the high and low OA and CLA groups. Functional enrichment analysis was performed on the top five differential hubbing genes, or for miRNAs based on the list of predicted target genes, to determine if these specific genes/miRNAs have biological relevance to fatty acid composition.

The regulatory impact factor (RIF) and phenotypic impact factor (PIF) scores were calculated as described in Reverter et al. (2010) to predict which transcripts were potential regulators of gene expression differences between the high and low OA and CLA groups. Regulatory impact factor 1 (RIF1) ranks genes as potential regulators of networks based largely on changes in correlations between two different states, while regulatory impact factor 2 (RIF2) ranks genes with more emphasis on how expression level changes between two different states (Reverter et al., 2010). Phenotypic impact factor (PIF) values were used to identify and rank genes based on the magnitude of gene expression and the difference in the expression of that gene between two treatments (Reverter et al., 2010). The RIF calculations presented here were modified from the original method, and the complete list of expressed mRNA or miRNA was tested as potential regulators, and only mRNAs or miRNAs with a significant partial correlation of 0.90 from PCIT were included in the RIF and PIF score estimates; as described in Cesar et al. (2016). PIF score estimates were ranked to select the top 10 PIF regulators of all genes in the dataset.

## Results

### Phenotypic and miRNA Expression Data

Genomic estimated breeding values (GEBVs) and the number of normalized mapped miRNA reads for Nelore steers genetically divergent for two FA content, oleic acid (C18:1 cis9) and conjugated linoleic acid (CLA-c9t11), are shown in **Table 1**. Sample sizes of extreme Nelore steers used for OA and CLA analyses were slightly different due to data availability. A Student’s *t*-test was previously performed by Cesar et al. (2016) to evaluate the mean differences between the high and low FA groups, and significant differences (*p*-value < 0.05) were observed for both OA and CLA content.

**Table 1:** Genomic estimated breeding values (GEBVs) and number of normalized mapped miRNA reads for Nelore steers with divergent fatty acid content groups.

Groups^1^	GEBVs	miRNA mapped reads	Groups^2^	GEBVs	miRNA mapped reads
**OA (C18:1 cis9)**					
H-OA1	2.11	970,350	L-OA1	−7.06	605,324
H-OA2	1.54	745,742	L-OA2	−4.98	1,308,497
H-OA3	3.20	1,009,408	L-OA3	−5.51	703,560
H-OA4	3.25	1,051,535	L-OA4	−2.41	525,015
H-OA5	2.16	1,052,657	L-OA5	−1.54	594,924
H-OA6	2.08	1,289,704	L-OA6	−2.95	563,260
H-OA7	1.58	610,678	L-OA7	−2.43	349,772
H-OA8	1.92	564,255	L-OA8	−7.03	557,486
H-OA9	4.14	555,136	L-OA9	−3.48	656,226
H-OA10	2.61	607,145	L-OA10	−2.67	685,506
H-OA11	3.89	1,035,882	L-OA11	−1.63	690,291
H-OA12	2.84	520,790	L-OA12	−8.07	748,090
H-OA13	1.76	584,448	L-OA13	−4.14	689,313
H-OA14	*	*	L-OA14	−7.36	602,604
H-OA15	*	*	L-OA15	−2.91	732,483
**Mean**	**2.16**	**745,742**	**Mean**	**−3.48**	**656,226**
**CLA (CLA-c9t11)**					
H-CLA1	0.008	439,844	L-CLA1	−0.008	457,706
H-CLA2	0.012	1,152,736	L-CLA2	−0.013	532,780
H-CLA3	0.008	938,908	L-CLA3	−0.017	988,632
H-CLA4	0.012	1308,497	L-CLA4	−0.011	521,652
H-CLA5	0.011	235,852	L-CLA5	−0.010	300,196
H-CLA6	0.016	874,538	L-CLA6	−0.009	1,264,789
H-CLA7	0.022	976,093	L-CLA7	−0.016	685,506
H-CLA8	0.025	1,039,805	L-CLA8	−0.013	702,016
H-CLA9	0.009	555,136	L-CLA9	−0.015	605,442
H-CLA10	0.009	918,555	L-CLA10	−0.013	677,737
H-CLA11	0.012	675,360	L-CLA11	−0.009	522,944
H-CLA12	0.012	772,395	L-CLA12	−0.010	658,923
H-CLA13	0.019	713,501	L-CLA13	−0.010	752,121
H-CLA14	0.019	500,697	L-CLA14	−0.018	1,306,312
H-CLA15	0.014	636,795	L-CLA15	−0.010	511,704
**Mean**	**0.012**	**772,395**	**Mean**	**−0.011**	**658,923**

^1^Nelore samples of high (H) oleic acid (OA) and conjugated linoleic acid (CLA) groups.

^2^Nelore samples of low (L) oleic acid (OA) and conjugated linoleic acid (CLA) groups.

*Data not available.

On average, 85% of miRNA reads for OA and CLA were mapped to the *B. taurus* UMD 3.1 genome assembly (Ensembl 84: Mar 2016). In total, 404 OA and 386 CLA mature miRNAs were detected by miRDeep2 software (**Supplementary Table 1**).

### Differentially Expressed miRNAs and Target Gene Identification

We identified 137 and 131 unique mature miRNA sequences with non-zero expression levels according to DESeq criteria for samples in OA (**Supplementary Table 2**) and CLA (**Supplementary Table 3**) groups, respectively, which were used in differential expression and co-expression analyses. The miRNAs bta-miR-126-5p and bta-miR-2419-5p were upregulated in the L-OA and H-CLA groups, respectively (**Table 2**).

**Table 2:** Differentially expressed miRNAs identified by miRDeep2 for Nelore steers with divergent fatty acid content groups and the number of predicted target genes.

miRNA	log2FC^1^	padj^2^	High^3^	Low^4^	Predicted target genes^5^
**OA (C18:1 cis9)**					
bta-miR-126-5p	0.4346	0.0987	3,334.235	4,682.251	3,619
**CLA (CLA-c9t11)**					
bta-miR-2419-5p	−1.18	0.0041	16,108.8	4,182.543	365

^1^log2 fold change.

^2^False discovery rate adjusted p-values by Benjamini–Hochberg (1995) methodology.

^3,4^Normalized miRNA mean counts of high and low fatty acid content groups.

^5^Number of predicted target genes by TargetsSan and miRanda software.

Among the 3,619 bta-miR-126-5p target genes (**Supplementary Table 4**) identified in the bovine genome, 162 were previously identified as DE in this same population (Cesar et al., 2016). *SCD* (stearoyl-CoA desaturase), *CDS2* (CDP-diacylglycerol synthase 2), *FAR2* (fatty acyl CoA reductase 2), and *NAB1* (NGFI-A binding protein 1) were included in this list of DE genes, well known to be related to biological processes associated with fatty acid composition. Regarding the 365 bta-miR-2419-5p target genes (**Supplementary Table 4**), 16 were also previously identified as DE (Cesar et al., 2016). Among these genes are included *CAV3* (caveolin 3), *JMJD1C* (jumonji domain containing 1C), *FOXO6* (forkhead box O6), and *PRKAG2* (protein kinase AMP-activated non-catalytic subunit gamma 2). These bta-miR-126-5p DE target genes were also enriched for genes involved in biological processes associated with fatty acid composition.

### miRNA and mRNA Co-Expression

#### Weighted Gene Co-Expression Network Analysis (WGCNA)

WGCNA was used to identify potential regulatory networks related to OA and CLA content in skeletal muscle. To this end, miRNA and mRNA expression data from animals with extreme GEBVs of OA and CLA were evaluated separately. After quality control, a total of 137 miRNAs and 16,710 mRNAs were analyzed for OA network construction, while a total of 131 miRNAs and 16,530 mRNAs were used for CLA network construction.

A total of 52 mRNA modules were identified in the H-OA group (**Figure S1A**), while in the L-OA group, 95 mRNA modules were identified (**Figure S1B**). Regarding miRNA, 12 and 9 modules were identified, respectively, in the H-OA (**Figure S2A**) and L-OA (**Figure S2B**) groups. For CLA, 52 and 28 mRNA modules in the H-CLA (**Figure S3A**) and L-CLA (**Figure S3B**) groups were identified, respectively. CLA miRNA network analysis identified six and five miRNA modules in the H-CLA (**Figure S4A**) and L-CLA (**Figure S4B**), respectively.

In order to investigate miRNA–mRNA interactions, mRNA module eigengenes (MEs), which represent the sum of gene expression profiles of each module, were correlated with miRNA MEs. The mRNA and miRNA modules with negative correlations and a nominal *p*-value < 0.10 were selected for further investigation. The focus on negative correlation between mRNA and miRNA modules was based on the fact that the predominant and canonical effect of miRNAs on gene expression is through mRNA downregulation, which would equate to a negative miRNA–mRNA expression correlation (Guo et al., 2010). In the H-OA group, four negative correlations between mRNA and miRNA MEs were identified, while in the L-OA group, eight negative correlations were observed (**Table 3**). In the H-CLA group, seven negative correlations between miRNA and mRNA MEs were observed, while in the L-CLA group, three negative correlations were observed (**Table 3**).

**Table 3:** Signaling pathways of miRNA module eigengenes (MEs) negatively correlated with mRNA MEs for Nelore steers with divergent fatty acid content groups.

Group	mRNA MEs	Corr	*p*-value^1^	miRNA MEs	Signaling pathways^2^	*p*-value^1^
**H-OA**	paletvioletred3	−0.7	0.01	Magenta	Insulin resistance	6.23e−06
	grey	−0.6	0.04	Black	MAPK signaling pathway	5.54e−07
	lightsteelblue1	−0.8	0.00	Turquoise	Insulin resistance	1.64e−04
	bisque4	−0.7	0.00			
**L-OA**	coral1	−0.7	0.00	Turquoise	AMPK signaling pathway	7.11e−07
	mediumpurple2	−0.6	0.01			
	darkolivegreen	−0.7	0.00	Pink	Insulin signaling pathway	9.76e−08
	orangered	−0.5	0.04			
	lightcyan1	−0.5	0.05	Blue	Proteoglycans in cancer	1.17e−04
	mediumorchid	−0.6	0.01			
	lightcyan1	−0.6	0.03	Yellow	Wnt signaling pathway	1.84e−05
	lightcyan1	−0.7	0.00	Green	Insulin signaling pathway	2.89e−04
**H-CLA**	cyan	−0.6	0.03	Turquoise	ns	*
	brown	−0.6	0.02	Red	Insulin resistance	7.9e−05
	plum2	−0.6	0.03	Blue	Insulin resistance	1.1e−05
	turquoise	−0.6	0.03			
	darkred	−0.7	0.00	Brown	ns	*
	steelblue	−0.5	0.04			
	thistle1	−0.6	0.02	Black	Insulin signaling pathway	2.73e−07
**L-CLA**	purple	−0.5	0.04	Blue	Insulin signaling pathway	6.68e−06
	white	−0.5	0.04			
	orange	−0.5	0.04	Red	MAPK signaling pathway	1.71e−04

^1^Nominal p-value.

^2^Signaling pathways of target genes from hub miRNAs selected based on greatest module membership values.

*NS, non-significant.

Hub miRNAs are the miRNAs with higher connectivity inside the module and are probably more informative (Filteau et al., 2013). In order to find hub miRNAs involved in the co-expression networks, we selected the top five miRNAs representing the greatest module membership (MM) values from miRNA modules negatively correlated with mRNA modules. **Table 3** shows signaling pathways obtained from WebGestalt software based on enrichment of the genes from miRNA modules and the target genes for hub miRNAs.

In the H-OA group, genes from mRNA modules were not significantly enriched for any Gene Ontology terms, while miRNA target genes from magenta and turquoise modules were significantly enriched for insulin resistance (**Supplementary Table 5**). miRNA target genes from black module were enriched for the MAPK signaling pathway (**Supplementary Table 5**). In the L-OA group, genes from the dark olive green module were significantly enriched for fatty acid degradation processes, while miRNA target genes from turquoise, pink, and blue modules were enriched for AMPK, insulin signaling pathway, and proteoglycans in cancer, respectively (**Supplementary Table 5**).

In the H-CLA group, genes from the mRNA modules brown and plum were significantly enriched for insulin resistance and steroid biosynthesis, respectively, while miRNA target genes from red, blue, and black modules were enriched for insulin resistance and insulin signaling pathway (**Supplementary Table 6**). In the L-CLA group, miRNA target genes from blue and red modules were enriched for insulin and MAPK signaling pathway, respectively (**Supplementary Table 6**).

**Figure 1:**
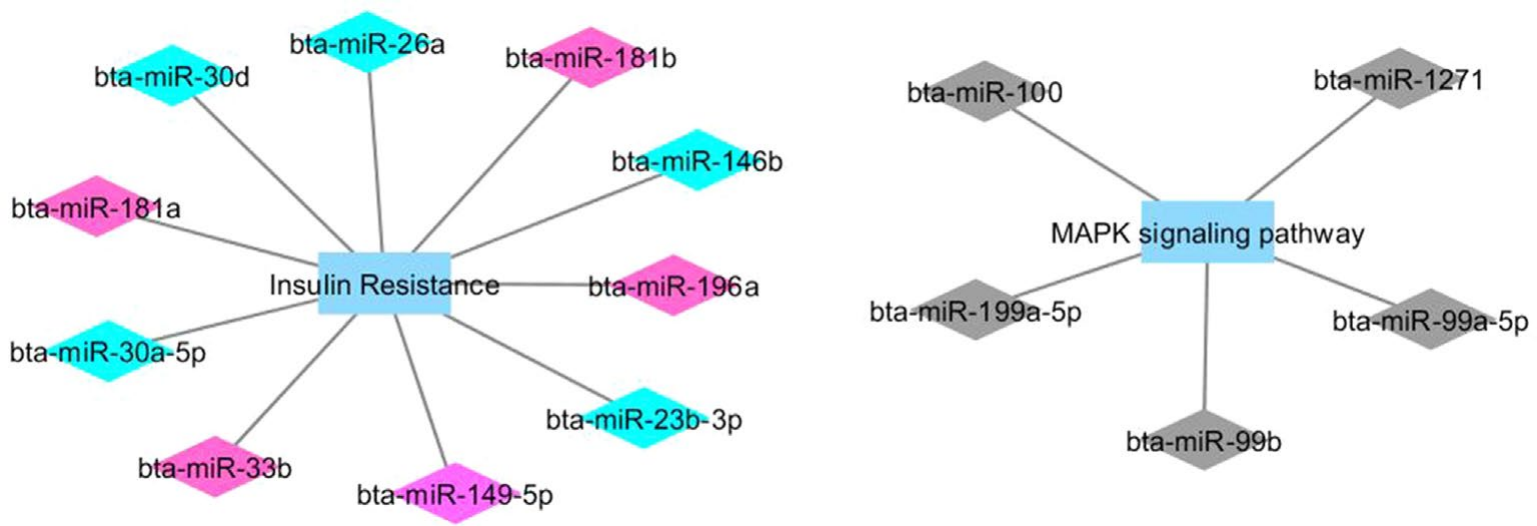
Co-expression networks of H-OA group from Nelore cattle. Colored diamonds represent the top five hub miRNAs within each module, and colored rectangles represent the signaling pathways associated (*p*-value ≤ 0.10) with hub miRNA target genes.

**Figure 2:**
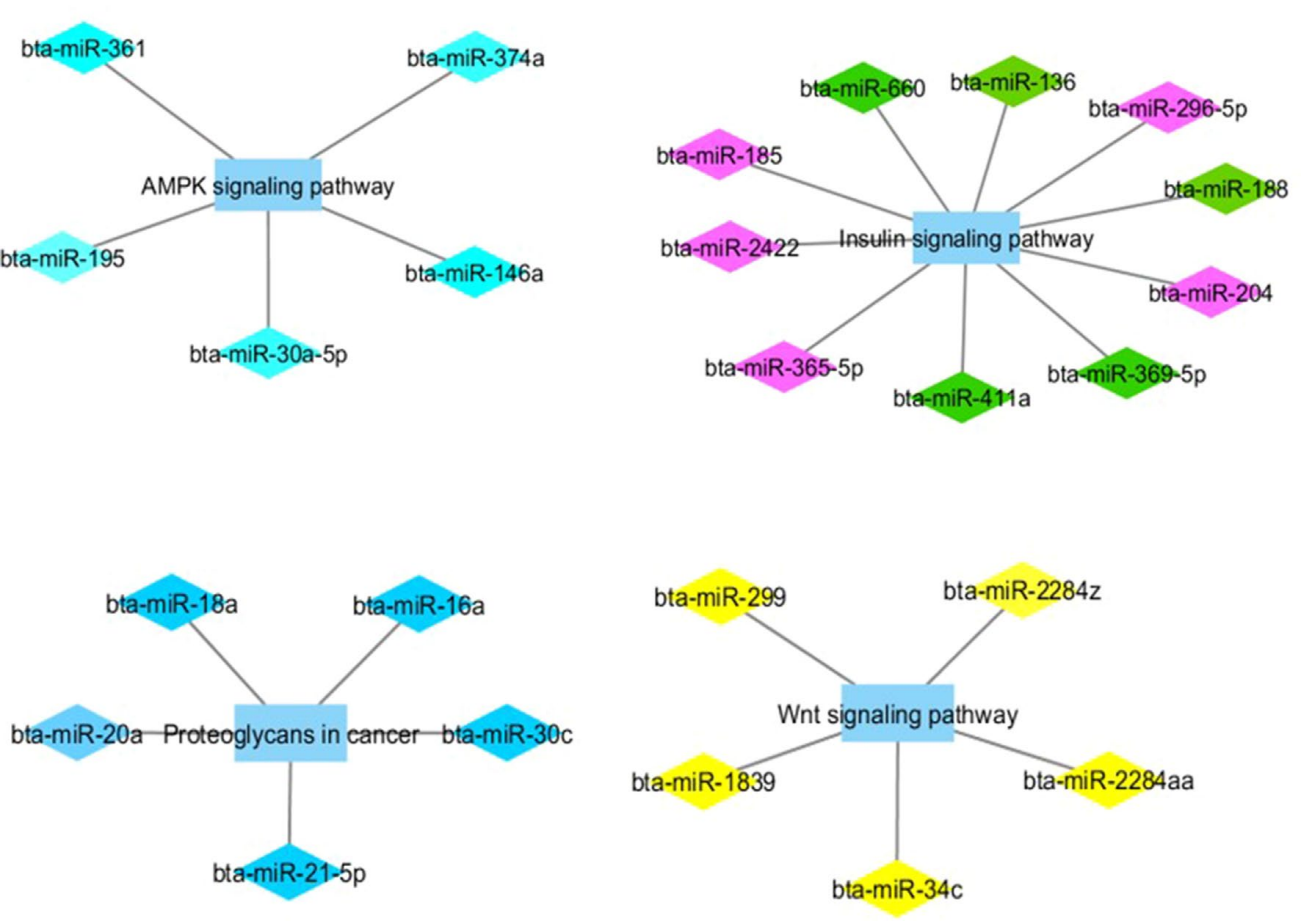
Co-expression networks of L-OA group from Nelore cattle. Colored diamonds represent the top five hub miRNAs within each module, and colored rectangles represent the signaling pathways associated (*p*-value ≤ 0.10) with hub miRNA target genes.

**Figure 3:**
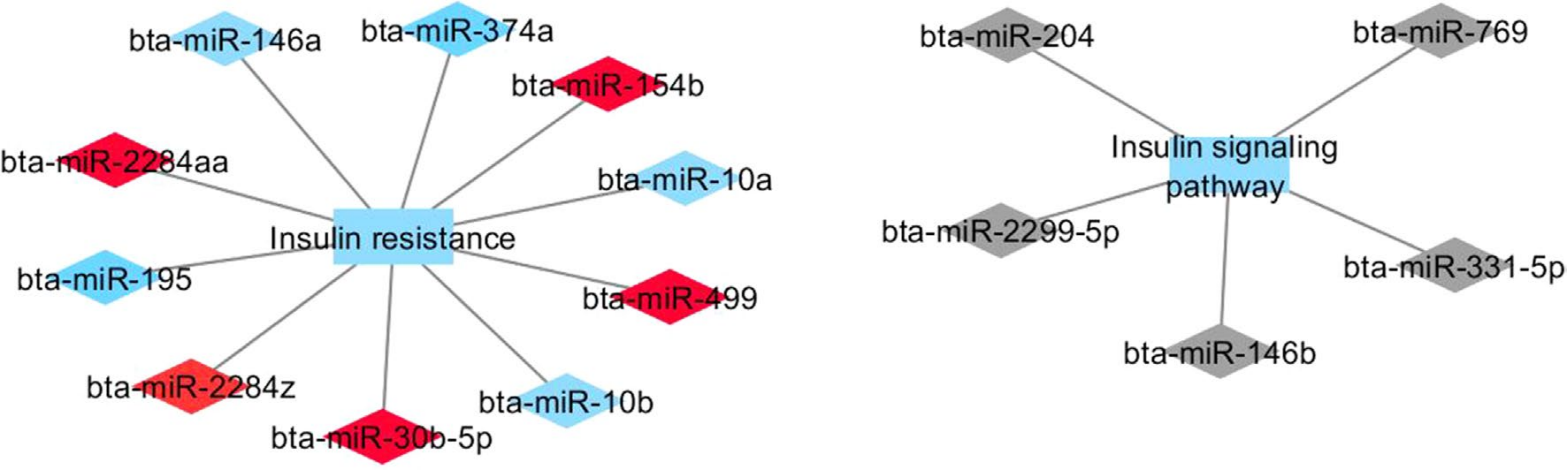
Co-expression networks of H-CLA group from Nelore cattle. Colored diamonds represent the top five hub miRNAs within each module, and colored rectangles represent the signaling pathways associated (*p*-value ≤ 0.10) with hub miRNA target genes.

**Figure 4:**
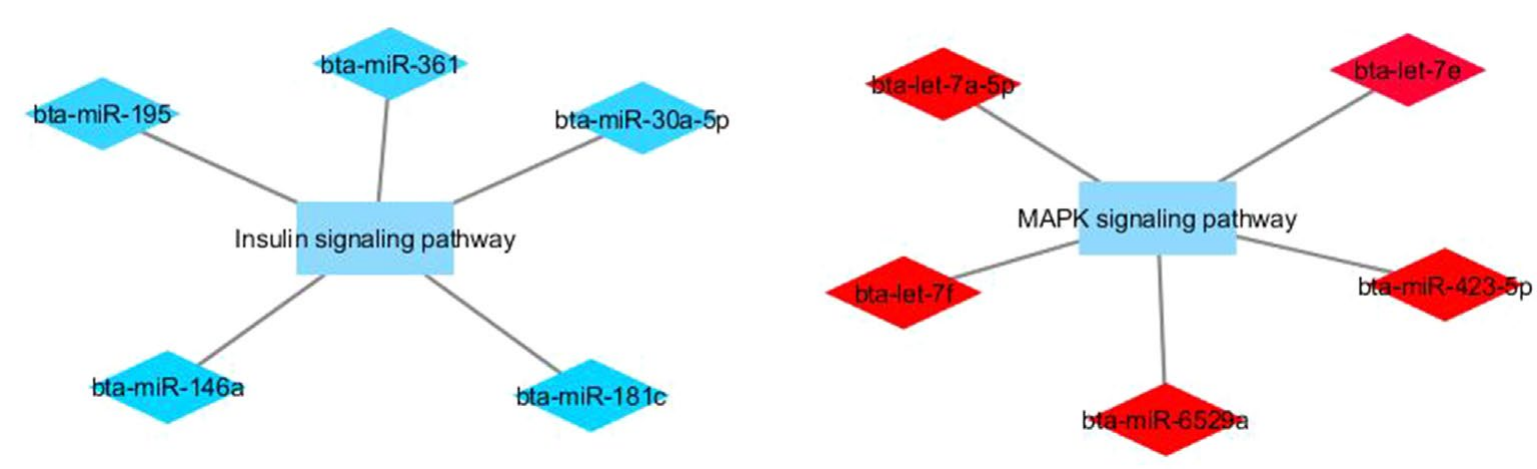
Co-expression networks of L-CLA group from Nelore cattle. Colored diamonds represent the top five hub miRNAs within each module, and colored rectangles represent the signaling pathways associated (*p*-value ≤ 0.10) with hub miRNA target genes.

**Figures 1** and **2** show co-expression networks for hub miRNAs enriched for signaling pathways related to FA composition in H-OA (**Figure 1**) and L-OA groups (**Figure 2**) and in H-CLA (**Figure 3**) and L-CLA (**Figure 4**) groups in Nelore cattle.

#### Partial Correlation With Information Theory (PCIT)

PCIT, regulatory impact factor (RIF), and phenotypic impact factor (PIF) were used to score genes as potential regulators of signaling pathways between high and low FA groups. Differential hubbing (DH) represents the number of significant partial correlations that a gene has between two phenotypic states (Hudson et al., 2009). DH values of all genes and miRNAs used in PCIT analysis are in **Supplementary Table 7**. **Table 4** shows the top five negative and positive extreme hubbing genes when comparing high and low OA and CLA groups. Functional enrichment analysis was performed on the top five DH genes to determine if they have biological relevance to fatty acid composition. The top 10 negatively and positively hubbed genes for OA and CLA content and their associated GO terms are shown in **Supplemental Table 8**.

**Table 4:** Top negative and positive extreme hubbing genes groups for oleic acid (OA) and conjugated linoleic acid (CLA) content.

Ensembl gene ID	Gene symbol	Description	DH*
**Negative hub genes OA **			
ENSBTAG00000040485	*THEG*	Theg spermatid protein	−905
ENSBTAG00000031806	*ZNF48*	Zinc finger protein 48	−826
ENSBTAG00000007692	–	–	−799
ENSBTAG00000044830	bta-mir-339a	bta-mir-339a	−770
ENSBTAG00000007956	*FRAT1*	Frequently rearranged advanced T-cell lymphomas 1	−753
**Positive hub genes OA**			
ENSBTAG00000015100	*ATP6V0E1*	ATPase H+ transporting V0 subunit e1	2946
ENSBTAG00000025221	TEX30	Testis expressed 30	2,925
ENSBTAG00000014337	*EIF2S3*	Eukaryotic translation initiation factor 2 subunit 3	2,911
ENSBTAG00000044066	*C1orf101*	Chromosome 1 open reading frame	2,903
ENSBTAG00000002772	*GAL3ST3*	Galactose-3-*O*-sulfotransferase 3	2,902
**Negative hub genes CLA**			
ENSBTAG00000005578	*KAT5*	Lysine acetyltransferase 5	−2,126
ENSBTAG00000046307	*CEBPD*	CCAAT enhancer binding protein delta	−2,112
ENSBTAG00000003849	*GNL2*	G protein nucleolar 2	−2,060
ENSBTAG00000045822	–	–	−1,997
ENSBTAG00000002634	*TMEM115*	Transmembrane protein 115	−1,987
**Positive hub genes CLA**			
ENSBTAG00000013600	*PSMG1*	Proteasome assembly chaperone	1,848
ENSBTAG00000015042	*WDR55*	WD repeat domain	1,809
ENSBTAG00000032684	*TCTEX1D2*	Tctex1 domain containing 2	1,794
ENSBTAG00000006321	*RTCA*	RNA 3′-terminal phosphate	1,740
ENSBTAG00000045544	*LOC107131189*	Eukaryotic translation initiation factor	1,692

*DH, differential hubbing.

The bta-mir-339a and *FRAT1* are among the top negatively hubbed genes for OA, associated with glycogen synthase kinase-3 binding protein (IPR008014) and Wnt signaling pathway (bta04310). *GAL3ST3* and *ATP6V0E1* are among the top positively hubbed genes, associated with the glycolipid biosynthetic process (GO: 0009247) and generation of metabolites and energy, respectively. *KAT5* is among the top negatively hubbed genes for CLA, associated with proteasome-mediated ubiquitin-dependent protein catabolic process (GO: 0043161), while *TMEM115* is associated with protein glycosylation (GO: 0006486). *PSMG1* is among the top positively hubbed genes and associated with proteasome assembly (GO: 0043248).

For OA, a total of 14,900 transcripts had negative RIF1 values, whereas 1,948 transcripts had positive values. For RIF2, 16,684 transcripts had negative values, while 127 transcripts had positive values (**Supplementary Table 9**). For CLA, a total of 14,171 transcripts had negative RIF1 values, while 1,891 transcripts had positive values. For RIF2, 108 transcripts had negative values, while 16,512 transcripts had positive values (**Supplementary Table 10**).

**Table 5** shows the top five negative and positive genes identified by RIF1 and RIF2 score by contrasting H and L groups for OA content. The RIF1 analysis identified putative regulators for OA content GO terms associated with muscling, e.g., muscle contraction and actin filament organization (*TPM1*, *TPM2*, and *MYL1*), and with fatness, e.g., glycolytic process and ATP biosynthetic process (*ALDOA*). The RIF2 analysis identified bta-mir-10b as a negative putative regulator, as well as the genes *ACTN2* and *TNNT1*, associated with skeletal muscle contraction GO terms. The same group of genes identified as positive RIF1 regulators is among the top positive RIF2 regulators. Functional enrichment analysis was performed using DAVID software on the top five genes to determine their biological relevance on fatty acid composition (**Supplementary Table 9**).

**Table 5:** Top negative and positive genes identified by regulatory impact factor 1 (RIF1) and regulatory impact factor 2 (RIF2) score for oleic acid (OA) content.

Ensembl gene ID	Gene symbol	Description	RIF1 *Z*-score*
**Top negative RIF1**			
ENSBTAG00000000020	*TRPV3*	Transient receptor potential cation channel subfamily V member 3	−0.046
ENSBTAG00000000030	*RDM1*	RAD52 motif containing 1	−0.046
ENSBTAG00000000102	*GPR75*	G protein-coupled receptor 75	−0.046
ENSBTAG00000000008	*KCNJ1*	Potassium voltage-gated channel subfamily J member 1	−0.046
ENSBTAG00000000168	–	–	−0.046
**Top positive RIF1**			
ENSBTAG00000012927	*ALDOA*	Aldolase, fructose-bisphosphate A	94.532
ENSBTAG00000011424	*TPM2*	Tropomyosin 2 (beta)	69.720
ENSBTAG00000013921	*CKM*	Creatine kinase	27.167
ENSBTAG00000009707	*MYL1*	Myosin light chain 1	23.357
ENSBTAG00000005373	*TPM1*	Tropomyosin 1 (alpha)	22.028
**Top negative RIF2**			
bta-miR-10b	bta-mir-10b	bta-mir-10b	−1.377
ENSBTAG00000007782	*MYOT*	Myotilin	−0.238
ENSBTAG00000009696	*ACTN2*	Alpha-actinin-2	−0.074
ENSBTAG00000000678	*CSDE1*	Cold shock domain containing E1	−0.060
ENSBTAG00000006419	*TNNT1*	Troponin T	−0.050
**Top positive RIF2**			
ENSBTAG00000012927	*ALDOA*	Aldolase, fructose-bisphosphate A	106.069
ENSBTAG00000011424	*TPM2*	Tropomyosin 2 (beta)	54.632
ENSBTAG00000013921	*CKM*	Creatine kinase	28.422
ENSBTAG00000005373	*TPM1*	Tropomyosin 1 (alpha)	22.117
ENSBTAG00000009707	*MYL1*	Myosin light chain 1	16.740

*RIF1 and RF2 scores are presented as Z-score-normalized values.

**Table 6** shows the top negative and positive RIF1 and RIF2 genes when high and low groups for CLA content were contrasted. RIF1 analysis identified putative negative regulators for CLA content genes related to regulation of gene expression (*GNRH1*) and muscling, e.g., actin filament organization (*TRPV4*). The genes *ALDOA*, *CKM*, and *TPM* were identified as positive RIF1 regulators and were also identified as negative RIF2 regulators. RIF2 analysis identified positive putative regulator genes enriched for GO terms associated with muscling (*MYH1*, *DES*, and *PDLIM3*). Functional enrichment analysis on the top five genes is presented in **Supplementary Table 10**.

**Table 6:** Top negative and positive genes identified by regulatory impact factor 1 (RIF1) and regulatory impact factor 1 (RIF2) score for conjugated linoleic acid (CLA) content.

Ensembl gene ID	Gene symbol	Description	RIF1 *Z*-score*
**Top negative RIF1**			
ENSBTAG00000000164	*GNRH1*	Gonadotropin releasing hormone 1	−0.0633
ENSBTAG00000000582	*LY6G6E*	Lymphocyte antigen 6 complex	−0.0633
ENSBTAG00000000982	*C22H3orf84*	Chromosome 22 c3orf84 homolog	−0.0633
ENSBTAG00000000031	*TRPV4*	Transient receptor potential cation channel subfamily V member 4	−0.0633
ENSBTAG00000000535	*PCNX2*	Pecanex homolog 2	−0.0633
**Top positive RIF1**			
ENSBTAG00000026986	*TTN*	Titin	25.8861
ENSBTAG00000006907	*NEB*	Nebulin	29.2850
ENSBTAG00000011424	*TPM2*	Tropomyosin 2 (beta)	36.2570
ENSBTAG00000013921	*CKM*	Creatine kinase	36.8083
ENSBTAG00000012927	*ALDOA*	Aldolase, fructose-bisphosphate A	89.4190
**Top negative RIF2**			
ENSBTAG00000012927	*ALDOA*	Aldolase, fructose-bisphosphate A	−108.25
ENSBTAG00000013921	*CKM*	Creatine kinase	−38.19
ENSBTAG00000011424	*TPM2*	Tropomyosin 2 (beta)	−38.08
ENSBTAG00000005373	*TPM1*	Tropomyosin 1 (beta)	−24.17
ENSBTAG00000046332	*ACTA1*	Actin alpha 1	−20.98
**Top positive RIF2**			
ENSBTAG00000018204	*MYH1*	Myosin heavy chain 1	11.5144
ENSBTAG00000005353	*DES*	Desmin	6.2929
ENSBTAG00000006907	*NEB*	Nebulin	4.5481
ENSBTAG00000006823	*CMYA5*	Cardiomyopathy associated 5	1.9259
ENSBTAG00000017183	*PDLIM3*	PDZ and LIM domain 3	1.3030

*RIF1 and RIF2 scores are presented as Z-score-normalized values.

For OA and CLA, PIF analysis identiﬁed 13,480 and 15,531 transcripts, respectively (adjusted *p*-value < 0.05), which could significantly impact fatty acid composition. **Table 7** lists the top 10 regulators ranked by PIF analysis for OA and CLA content. Functional enrichment analysis to determine the biological relevance on fatty acid composition of the top 10 genes is presented in **Supplementary Table 11**.

**Table 7:** Top 10 regulators identiﬁed by phenotypic impact factor (PIF) analysis with FDR adjusted *p*-value for oleic acid (OA) and conjugated linoleic acid (CLA) content.

Ensembl gene ID	Gene symbol	Description	PIF	adj.*p*.Val
**OA**				
bta-miR-10b	bta-miR-10b	bta-miR-10b	66,197	1.10e−01*
ENSBTAG00000046332	*ACTA1*	Actin, alpha 1, skeletal muscle	20,402	1.27e−005
ENSBTAG00000026986	*TTN*	Titin	8,820	4.81e−007
ENSBTAG00000018204	*MYH1*	Myosin, heavy chain 1, skeletal muscle, adult	7,401	8.64e−008
ENSBTAG00000012927	*ALDOA*	Aldolase, fructose-bisphosphate A	7,199	9.68e−006
ENSBTAG00000011424	–	–	4,547	2.42e−005
ENSBTAG00000013921	*CKM*	Creatine kinase, M-type	3,610	5.41e−006
bta-miR-486	bta-miR-486	bta-mir-486	3,506	4.27e−001*
ENSBTAG00000006907	*NEB*	Nebulin	2,864	1.91e−007
ENSBTAG00000021218	*MYLPF*	Myosin light chain, phosphorylatable, fast skeletal muscle	2,111	6.31e−005
**CLA**				
bta-miR-10b	bta-miR-10b	bta-miR-10b	92,224	9.53e−003
ENSBTAG00000046332	*ACTA1*	Actin, alpha 1, skeletal muscle	39,297	1.48e−009
ENSBTAG00000012927	*ALDOA*	Aldolase, fructose-bisphosphate A	21,787	7.32e−004
ENSBTAG00000026986	*TTN*	Titin	18,487	2.16e−007
ENSBTAG00000018204	*MYH1*	Myosin, heavy chain 1, skeletal muscle, adult	14,134	1.65e−011
ENSBTAG00000011424	–	–	9,845	2.65e−004
ENSBTAG00000013921	*CKM*	Creatine kinase, M-type	7,206	1.41e−006
ENSBTAG00000006907	*NEB*	Nebulin	5,455	6.68e−007
ENSBTAG00000021218	*MYLPF*	Myosin light chain, phosphorylatable, fast skeletal muscle	4,229	1.27e−004
bta-mir-486	bta-mir-486	bta-mir-486	3,777	1.18e−003

*Non-significant adj p-values.

Interestingly, among the top 10 regulators identiﬁed by PIF analyses for OA and CLA content is the same group of genes (*ACTA1*, *TTN*, *MYH1*, *ALDOA*, *CKM*, and *NEB*) with Gene Ontology (GO) terms associated with muscling (*ACTA1*, *MYH*, and *MYLPF*) and fatness and fiber type (*ALDOA*).

## Discussion

The purpose of this study was to investigate the complex interactions between miRNAs and mRNAs in bovine skeletal muscle associated with variation in oleic acid (OA) and conjugated linoleic acid (CLA-c9t11) content. This was accomplished by performing differential miRNA expression analysis and two different gene co-expression approaches, weighted gene co-expression network analysis (WGCNA) and partial correlation with information theory (PCIT). Furthermore, we sought to identify the key drivers of gene expression networks. Analyses were focused on these two fatty acids due to their importance in many biological processes and beneficial effects on metabolic diseases and human health (Laaksonen et al., 2005b). Also, a significant number of differentially expressed genes in response to OA (1134) and CLA (872) content were identified in this same population previously (Cesar et al., 2016). OA is a monounsaturated fatty acid present in membrane phospholipids, triglycerides, and cholesterol and is associated with protection against heart disease (Laaksonen et al., 2005b), while CLA antidiabetic effects are mediated *via* anti-inflammatory processes in white adipose tissue (Moloney et al., 2007).

Integration of miRNA and mRNA co-expression from next-generation sequencing data from the same individuals revealed the possible regulatory roles of miRNAs on fatty acid composition. WGCNA allowed the identification of miRNA and mRNA modules that were negatively correlated with each other, which may indicate that FA composition is modulated by specific miRNA–mRNA interactions. However, only one mRNA module in the L-OA group and two mRNA modules in the H-CLA group presented functional enrichment for fatty acid degradation, insulin resistance, and steroid biosynthesis. Therefore, the subsequent analyses were focused on the identification of key miRNAs that may be involved in co-expression networks and thereby in the regulation of fatty acid composition.

From differential expression analysis, the *SCD* gene, identified as a putative target gene of bta-miR-126-5p, was found to be upregulated in the H-OA content group in a previous skeletal muscle RNAseq study (Cesar et al., 2016). *SCD* is as a key enzyme in *de novo* lipogenesis (Scaglia and Igal, 2005), and its upregulation has been associated with deposition of unsaturated FAs. Thus, the upregulated bta-miR-126-5 in the L-OA group observed herein may explain the reduced *SCD* mRNA levels observed before (Cesar et al., 2016). These results suggest that *SCD* expression level is related to OA content and may suggest a new role for bta-miR-126-5p in the regulation of *SCD*. It is relevant to note that only negative miRNA–mRNA correlations were considered herein. However, it is important to emphasize that this was an exploratory study and should be complemented by other *in vitro* and *in vivo* analyses to better discriminate the mechanisms of miRNA regulation of gene expression.

Functional enrichment analysis indicated a relationship between insulin, insulin resistance, adipocytokine signaling pathway, and non-alcoholic fatty liver disease for *PRKAG2* gene, a target gene of bta-miR-2419-5p. The major effects of insulin on muscle and adipose tissue are related to carbohydrate, lipid, and protein metabolism (Dimitriadis et al., 2011). Insulin can decrease the rate of lipolysis, stimulate fatty acid synthesis, increase the uptake of triglycerides from blood into muscle and adipose tissue, and decrease the rate of fatty acid oxidation in muscle and liver (Dimitriadis et al., 2011). Metabolic diseases such as obesity and coronary disorders can be the consequence of insulin resistance, i.e., the inability of insulin to drive glucose into muscle and other tissues, which can be caused by excessive body fat deposition (Dimitriadis et al., 2011). Assuming that bta-miR-2419-5p expression level regulates CLA content and that this miRNA can regulate *PRKAG2* expression, it is possible that bta-miR-2419-5p can regulate insulin expression to ultimately respond to CLA content. The transcription factors (TFs) *FOXO1*, *FOXO3*, and *FOXO4* are also targets of bta-miR-2419-5p. Therefore, they may also be regulated by CLA content. *FOXO1* expression has been previously associated with CLA content in this population (Cesar et al., 2016).

Co-expression network analysis integrating miRNA and mRNA expression data revealed two miRNA modules (magenta and turquoise) in the H-OA group whose potential target genes were significantly enriched for the GO term insulin resistance. As discussed previously, it is known that insulin resistance can be caused by excessive fat deposition (Ortega et al., 2013). The miRNA magenta module grouped both bta-miR-181a and bta-miR-33a/b. The bta-miR-181a is from the same family as bta-miR-181b, which has been reported to regulate the biosynthesis of bovine milk by targeting *ACSL1*, an important enzyme of milk lipid synthesis (Lian et al., 2016). Furthermore, bta-miR-33a/b has been reported to contribute to the regulation of fatty acid metabolism and the insulin signaling pathway (Dávalos et al., 2011), which indicates that the insulin signaling pathway may be impacted in the H-OA group.

The miRNA turquoise module contained both bta-miR-146b and bta-miR-26b. In a recent integrative analysis of miRNA–mRNA expression related to intramuscular fat (IMF) deposition in animals from this population, the bta-miR-146b was found to be downregulated in individuals with high IMF content, while the bta-miR-26b was identified by PCIT as a candidate regulatory gene that negatively regulates IMF deposition (Oliveira et al., 2018). IMF represents the amount of fat accumulated between muscle fibers or within muscle cells, and it is a determinant factor that affects meat quality (Wood et al., 2008). Despite the fact that in the present study IMF deposition between high and low groups was not statistically different (Cesar et al., 2016), we could not ignore the fact that OA, CLA, and other FAs comprise IMF. It has been reported that as intramuscular lipid content accumulates, there is a concomitant elevation in the concentration of oleic acid (Smith et al., 2006). Other hub miRNAs such as bta-miR-196a and bta-miR-30f (from the same family of hubs bta-miR-30d and bta-miR-30a-5p) have been reported to have a higher expression level in cattle that have higher amounts of IMF (Guo et al., 2017). Therefore, taking together the results from these and previous studies, we can highlight the role of bta-miR-146b, bta-miR-26b, bta-miR-30d, and bta-miR-196a in regulating muscle fatty acid composition.

Lipid accumulation can activate the immune system and inflammatory pathways due to the secretion of proinflammatory molecules by adipocytes (Lau, 2005). The mitogen-activated protein kinase (MAPK) cascade, enriched from the miRNA black module, is highly conserved and involved in various cellular functions, including responses to proinflammatory stimuli (Soares-Silva et al., 2016). Networks and DE genes related to immune system and inflammatory response were previously associated with high amounts of IMF in this population (Cesar et al., 2015; Oliveira et al., 2018). Among the hub miRNAs from the black module was the bta-miR100, which was previously found to be downregulated in animals with high IMF (Oliveira et al., 2018).

In the L-OA group, the target genes of two miRNA modules (pink and green) were enriched for GO terms associated with the insulin signaling pathway. Insulin is a hormone with a direct effect on lipid metabolism (Dimitriadis et al., 2011), which could explain why modules present in the high and low fatty acid groups are associated with insulin-related terms. However, we can observe an overrepresentation of the insulin signaling pathway in the low fatty acid group, while in the high fatty acid group, we can observe an overrepresentation of the insulin resistance pathway. As discussed before, insulin resistance has been linked to excessive body fat deposition and obesity (Dimitriadis et al., 2011), supporting our findings.

Hub miRNAs from the pink module (bta-miR-204 and bta-miR-365-5p) and from the green module (bta-miR-660) have been previously associated with adipose tissue in cattle (Gu et al., 2007). Other hub miRNAs from the green module (bta-miR-411a and bta-miR-136) were expressed at a higher level in Wagyu compared with Holstein cattle (Guo et al., 2017). Wagyu cattle accumulate large amounts of marbling and specifically monounsaturated fatty acids, of which oleic acid is primarily responsible for the soft fat (Smith et al., 2009). Taken together, these results may indicate a possible role of these miRNAs in post-transcriptional regulation of OA content.

Target genes of the miRNA turquoise module were enriched for GO terms associated with the AMPK signaling pathway. *AMPK* is a basic regulator of cellular and body energy metabolism and may enhance activity of mitochondrial proteins involved in oxidative metabolism (Thomson et al., 2008). Cesar et al. (2016) reported that several canonical pathways of oxidative phosphorylation were upregulated in animals with H-OA content. Therefore, the enrichment of target genes associated with the AMPK signaling pathway in L-OA might indicate a post-transcriptional regulation of this pathway resulting in downregulated oxidative metabolism in these animals, complementing the findings by Cesar et al. (2016). Still on the turquoise module, the hub bta-miR-146b, which is in the same miRNA family as bta-miR-146a, has been correlated with target genes that are functionally enriched for GO terms associated with fatty acid oxidation (Oliveira et al., 2018).

Target genes of the miRNA blue module were enriched for GO terms associated with proteoglycans in cancer pathways. Proteoglycans have been shown to be key macromolecules that contribute to biology of various types of cancer (Iozzo and Sanderson, 2011). Previous studies have reported an important contribution of OA intake to human health, with protective effects against cancer development (Schwartz et al., 2008). In this sense, genes related to cancer were found to be upregulated in animals with low CLA content (Cesar et al., 2016). These findings may be evidence of miRNA modulation of a carcinogenic pathway. In the miRNA blue module as well, a hub miRNA, bta-miR-21-5p, has been reported to be an important regulator of bovine mammary lipogenesis and metabolism (Li et al., 2015). As in meat, fatty acid composition can influence the nutritional quality of milk and milk fat (Soyeurt et al., 2008). From miRNA yellow module, target genes were enriched for Wnt signaling pathway. Wnt is a member of signal transduction pathways, which regulates crucial aspects of cell fate determination (Komiya and Habas, 2008). It has been reported that the knockdown of a key enzyme in fatty acid synthesis (*FASN*) could attenuate the Wnt signaling pathway *via* downregulation of specific genes (Wang et al., 2016).

Two miRNA modules (red and blue) identified in the H-CLA group were enriched for GO terms associated with insulin signaling pathway and insulin resistance. The hub bta-miR-30b-5p from red module has been reported to regulate muscle cell differentiation (Zhang et al., 2016), while hub bta-miR-10b from blue module and bta-miR-146b from grey module have been reported to have a higher expression in mammary tissue (Wicik et al., 2016). Moreover, bta-miR-146b was associated with the GO terms of Wnt and inﬂammatory pathways. The canonical Wnt signaling pathway was also enriched in the L-OA group.

In the L-CLA group, target genes in the miRNA blue module were enriched for the insulin signaling pathway and target genes of the miRNA red module for MAPK signaling pathway GO terms. Besides being involved with inflammatory processes, the MAPK signaling pathway is involved in the activation of PPARα (peroxisome proliferator-activated receptor) by adiponectin, stimulating fatty acid oxidation in muscle cells (Myeong et al., 2006). Adiponectin is an adipocytokine secreted by adipocytes with its beneﬁcial effects on insulin resistance and metabolic disorders (Myeong et al., 2006). Interestingly, hub miRNAs from the red module such as bta-let-7a-5p, bta-let-7f, and bta-let-7e have been associated with insulin-like growth factor receptor signaling pathway (Wicik et al., 2016).

To better understand the biological processes that influence muscle FA composition, PCIT analysis was conducted. In this analysis, we identified the potential regulators that could be involved in the gene expression changes in skeletal muscle due to OA and CLA content. Negative and positive regulators are defined based on the number of significant partial correlations that a gene has between two states (Reverter and Chan, 2008).

Differential hubbing (DH) analysis identified bta-mir-339a as one of the top five negative regulators, with more connections in the L-OA group. The target genes of bta-mir-339a were enriched for 10 significant pathways, including the MAPK signaling pathway, which was also identified by WGCNA. Furthermore, bta-mir 339 has been reported to be expressed at a higher level in bovine adipose tissue than in other tissues (Gu et al., 2007). DH analysis also identified the *FRAT1* gene as a potential negative regulator, which is associated with the Wnt signaling pathway. As discussed previously, the Wnt signaling pathway would be affected by the knockdown of *FASN*, resulting in lower fatty acid synthesis, which complements our findings from WGCNA in the L-OA group. Among the top 10 positive differentially hubbed genes is the *ATP6V0E* gene, from the same family of *ATP6V1D* gene, which has been reported as a factor mediating hepatic steatosis (Nakadera et al., 2016), a metabolic syndrome frequently associated with obesity and diabetes. Furthermore, the *GAL3ST3* gene has been associated with lipid biosynthetic processes (Suzuki et al., 2001).

For CLA content, DH analysis identified *KAT5* as extreme negative and *PSMG1* as extreme positive hubbed genes associated with GO terms for proteasome pathways. The proteasome is a large protein complex responsible for degradation of intracellular proteins (Tanaka, 2009), and proteasome dysfunction has been associated with oxidative stress and insulin sensitivity in human obesity (Díaz-Ruiz et al., 2015). Gonçalves et al. (2018) otherwise concluded that the proteasome pathway may be a potential regulator of beef tenderness in this population. The total lipid content of muscle has a recognized role in beef tenderness, and the concentration of fatty acids is positively correlated with the palatability of beef (Wood et al., 2008). The proteasome pathway was also previously associated with OA content in this population (Cesar et al., 2016), supporting our findings of genes related to proteasome pathways as potential regulators.

Bta-miR-10b was identiﬁed as a CLA candidate regulator by both RIF2 and PIF analyses, being also pointed as a hub miRNA by WGCNA. Although its target genes were not enriched for any pathways specifically related to fatty acid metabolism, this miRNA has been previously correlated with backfat thickness and adipose tissue in cattle (Gu et al., 2007; Jin et al., 2010). Bta-mir-486, which was identified by PIF analysis as a top regulator for CLA content, has been associated with skeletal muscle growth (Jing et al., 2015) and was recently found to be downregulated in feed efficient animals of this same cattle population (De Oliveira et al., 2018).

*ACTA1* is the gene with the highest PIF rank, which indicates that it may be the most important gene related to both OA and CLA variations in this population. *ACTA1* expression is specific to muscle fibers, with an essential role in muscle contraction and cell morphology (Pollard and Cooper, 1986). *ALDOA* gene was the fourth and second major regulator identified by RIF and PIF analyses for OA and CLA content, respectively. This gene is involved in adipogenic differentiation, which is critical for intramuscular fat deposition and meat quality (Li et al., 2016). The same group of genes (*TPM2*, *CKM*, *TPM1*, and *MYL1*), including *ALDOA*, was identified as positive RIF1 and RIF2 regulators for OA content, indicating the relevance of these genes in fatty acid metabolism. Moreover, Oliveira et al. (2018) also identified the *ALDOA* gene as a putative regulatory for the differences in IMF deposition, which, taken together, may indicate that *ALDOA* is an important gene regulator of fatty acid deposition and composition in Nelore cattle.

In this integrative analysis, insulin resistance, insulin, and MAPK signaling pathways were overrepresented in high and low fatty acid groups. These signaling pathways have been linked to adipocyte differentiation and lipogenesis in cattle (Guo et al., 2017). Based on the literature and our results, insulin and inflammatory processes are influencing OA and CLA composition in Nelore cattle. This study also indicates that hub miRNAs like bta-miR-33a/b, bta-miR-100, bta-miR-204, bta-miR-365-5p, bta-miR-660, bta-miR-411a, bta-miR-136, bta-miR-30-5p, bta-miR-146b, bta-let-7a-5p bta-let-7f, and bta-let-7e are involved with these biological processes. Among the results pointed out by both RIF and PIF analyses, the bta-mir 339, bta-mir-10b, bta-miR 486, and genes *ACTA1* and *ALDOA* are the most relevant regulators for muscle fatty acid composition in Nelore cattle.

Fat and fatty acids, whether in adipose tissue or muscle, contribute to various aspects of meat quality and are central to the nutritional value of meat (Wood et al., 2008). Furthermore, they can have beneficial effects on human health. OA consumption is associated with low levels of low-density lipoprotein (LDL) or “bad cholesterol,” which in turn may reduce atherosclerosis risk and diabetes occurrence. Further, OA consumption could increase levels of high-density lipoprotein (HDL) in blood (Smith et al., 2009), whereas CLA consumption may contribute to reduced body fat, cardiovascular diseases, and cancer and can modulate inflammatory responses (Dilzer and Park, 2012).

## Conclusions

In the present study, signaling pathways, miRNAs, and gene regulators related to fatty acid composition in Nelore cattle were identified by miRNA expression and gene co-expression network approaches. Although some of these potential regulators have been previously linked to fatty acid composition, the complex miRNA–mRNA regulatory network has never been reported so far. This study improves our understanding of the molecular mechanisms controlling intramuscular muscle fat composition in bovines, revealing new candidate networks regulating OA and CLA phenotypes, which could positively benefit beef production.

## Ethics Statement

Experimental procedures were carried out in accordance with the relevant guidelines provided by the Institutional Animal Care and Use Committee Guidelines of the Embrapa Pecuria Sudeste—Protocol CEUA 01/2013. The Ethical Committee of the Embrapa Pecuria Sudeste (Sao Carlos, Sao Paulo, Brazil) approved all experimental protocols (approval code CEUA 01/2013) prior to the conduction of the study.

## Author Contributions

PO, LC, and LR conceived and designed the experiment; PO, LC, AC, GM, AZ, and LC performed the experiments; PO, AC, WD, and MS performed analysis; PO, AC, WD, BA, JK, JR, and LR interpreted the results; PO, AC, WD, MS, BA, JK, JR, and LR drafted and revised the manuscript. All authors read and approved the final manuscript.

## Funding

This study was conducted with funding from EMBRAPA, São Paulo Research Foundation (FAPESP, grant number: 2012/23638-8), and scholarship to PO (grant numbers: 2014/22235-1 and 2016/03291-4), the National Council for Scientific and Technological Development (CNPq, grant numbers: 449172/2014-7, 303754/2016-8, 444374/2014-0, and 309004/2016-0), and fellowships to LR, LC, and GM.

## Conflict of Interest Statement

The authors declare that the research was conducted in the absence of any commercial or financial relationships that could be construed as a potential conflict of interest.

## Abbreviations

CLA, conjugated linoleic acid; DH, differential hubbing; FA, fatty acid; GEBV, genomic estimated breeding value: IMF, intramuscular fat content: ME, module eigengene: MM, module membership: OA, oleic acid: PCIT, partial correlation with information theory: PIF, phenotypic impact factor: RIF1, regulatory impact factor 1: RIF2, regulatory impact factor 2: WGCNA, weighted gene co-expression network analysis:
