# Supplementary material for: Co-Expression Networks Reveal Potential Regulatory Roles of miRNAs in Fatty Acid Composition of Nelore Cattle
Source: Front Genet. 2019 Jul 11;10:651. doi: 10.3389/fgene.2019.00651 (PMC6637853; doi:10.3389/fgene.2019.00651)

Co-expression networks reveals potential regulatory roles of miRNAs in fatty acids composition of Nelore cattle.

Priscila SN de Oliveira[1], Luiz L Coutinho[2], Aline SM Cesar[3], Wellison J da S Diniz[4], Marcela M de Souza[5], Bruno G Andrade[1], James E Koltes[5], Gerson B Mourão[2], Adhemar Zerlotini[6], James M Reecy[5] and Luciana CA Regitano[*].

Supplementary Figure Information

Fig S1. Cluster dendrogram and module assignment for high (a) and low (b) Oleic Acid group of mRNA modules from WGCNA.

a)

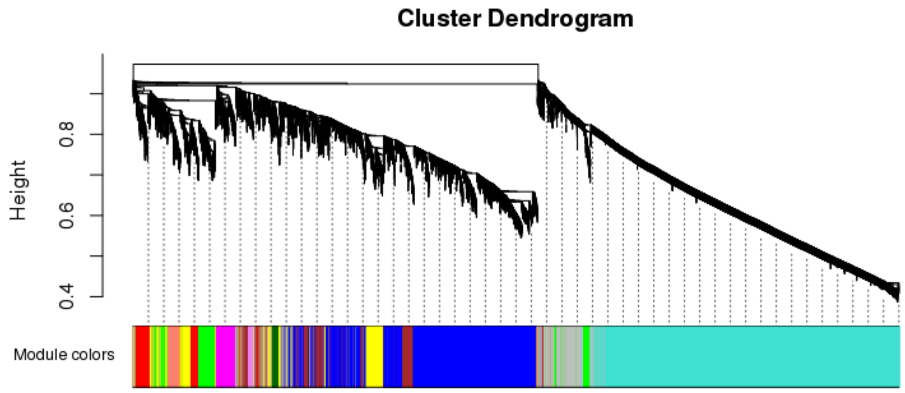

b)

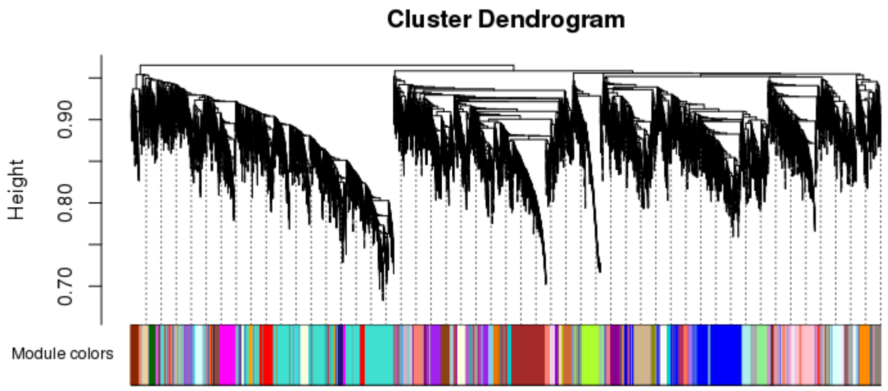

Fig S2. Cluster dendrogram and module assignment for high (a) and low (b) Oleic Acid group of miRNA modules from WGCNA.

a)

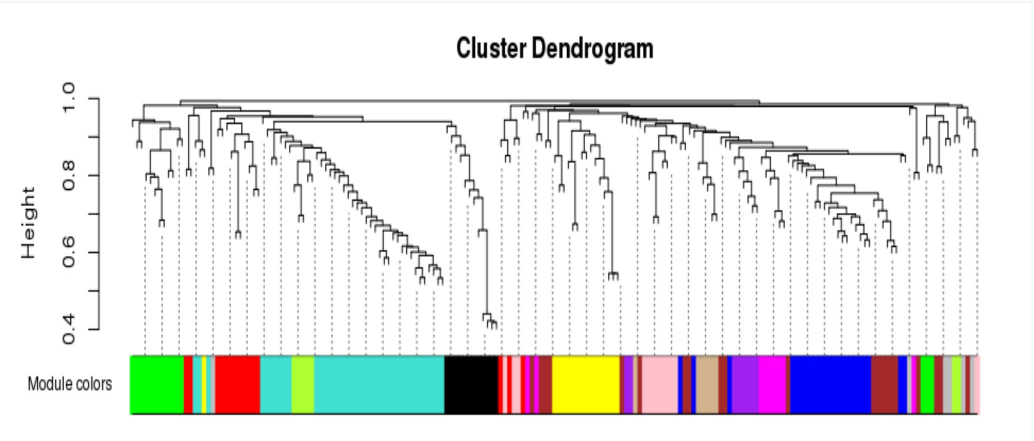

b)

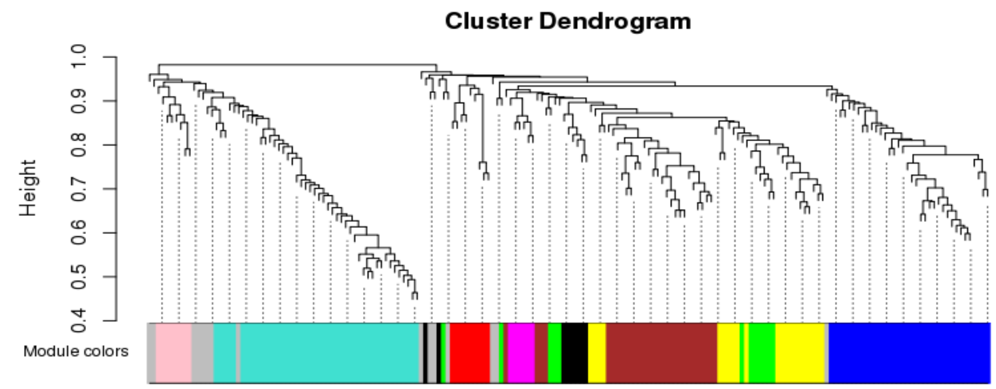

Fig S3. Cluster dendrogram and module assignment for high (a) and low (b) Conjugated Linoleic Acid group of mRNA modules from WGCNA.

a)

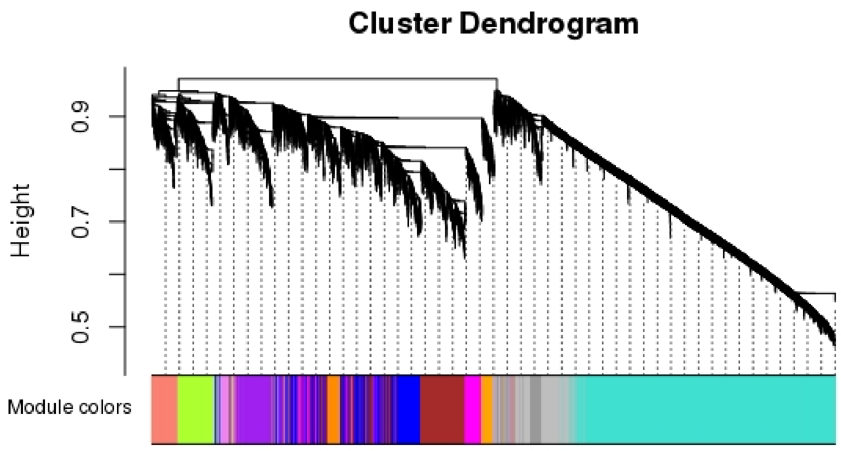

b)

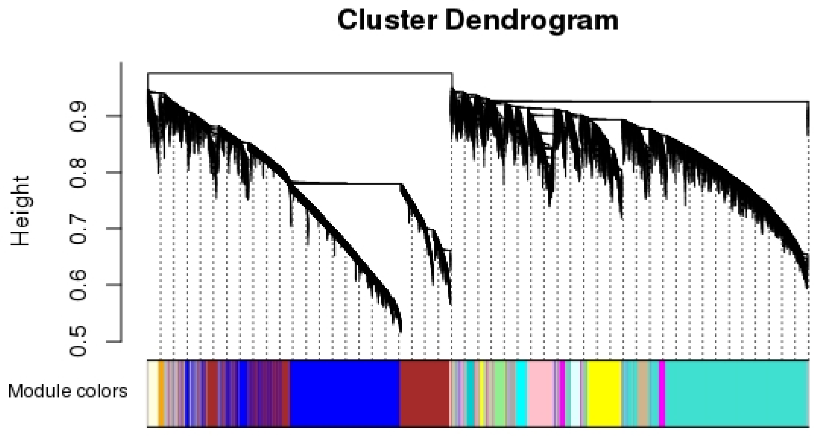

Fig S4. Cluster dendrogram and module assignment for high (a) and low (b) Conjugated Linoleic Acid group of miRNA modules from WGCNA.

a)

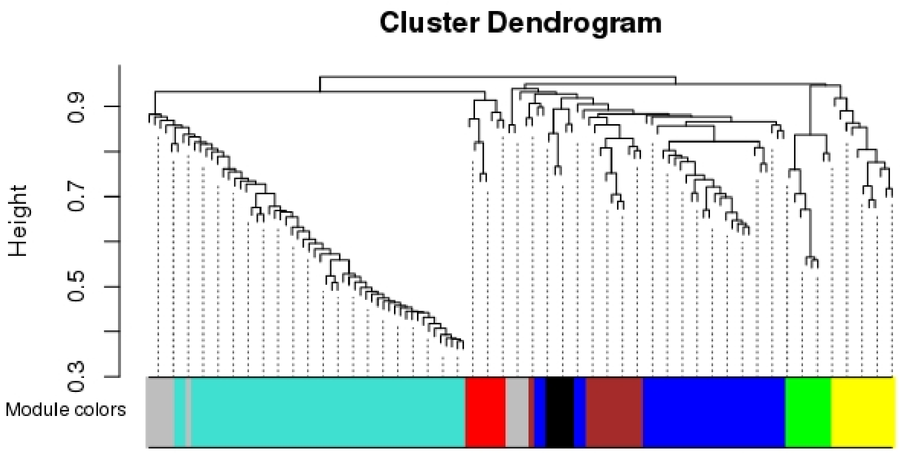

b)

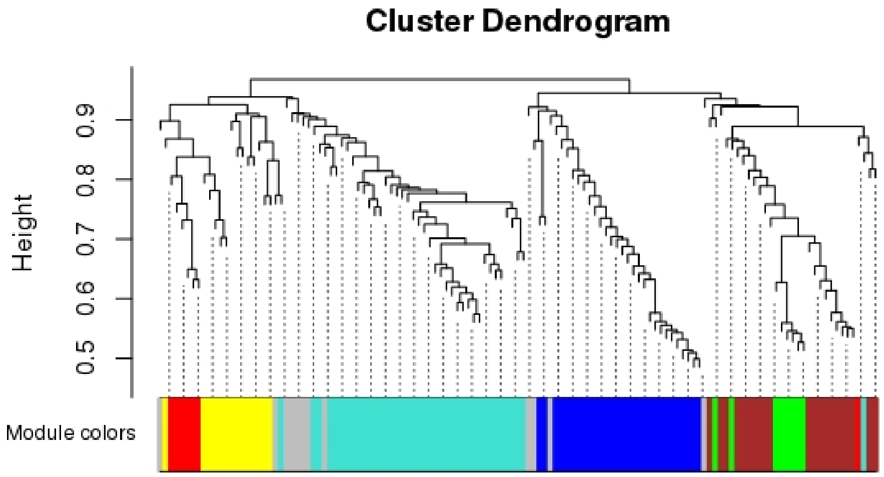

Supplement: Supplementary file 12 [file DataSheet_1.pdf]
